# A biopsychosocial model based on negative feedback and control

**DOI:** 10.3389/fnhum.2014.00094

**Published:** 2014-02-28

**Authors:** Timothy A. Carey, Warren Mansell, Sara J. Tai

**Affiliations:** ^1^Centre for Remote Health, a joint Centre of Flinders University and Charles Darwin UniversityAlice Springs, NT, Australia; ^2^Central Australian Mental Health Service, NT Department of Health and FamiliesAlice Springs, NT, Australia; ^3^School of Psychological Sciences, University of ManchesterManchester, UK

**Keywords:** negative feedback, biopsychosocial model, control, mechanism, control system

## Abstract

Although the biopsychosocial model has been a popular topic of discussion for over four decades it has not had the traction in fields of research that might be expected of such an intuitively appealing idea. One reason for this might be the absence of an identified mechanism or a functional architecture that is authentically biopsychosocial. What is needed is a robust mechanism that is equally important to biochemical processes as it is to psychological and social processes. Negative feedback may be the mechanism that is required. Negative feedback has been implicated in the regulation of neurotransmitters as well as important psychological and social processes such as emotional regulation and the relationship between a psychotherapist and a client. Moreover, negative feedback is purported to also govern the activity of all other organisms as well as humans. Perceptual Control Theory (PCT) describes the way in which negative feedback establishes control at increasing levels of perceptual complexity. Thus, PCT may be the first biopsychosocial model to be articulated in functional terms. In this paper we outline the working model of PCT and explain how PCT provides an embodied hierarchical neural architecture that utilizes negative feedback to control physiological, psychological, and social variables. PCT has major implications for both research and practice and, importantly, provides a guide by which fields of research that are currently separated may be integrated to bring about substantial progress in understanding the way in which the brain alters, and is altered by, its behavioral and environmental context.

The biopsychosocial model in psychology (Engel, 1980) was suggested with great promise as a viable and much needed alternative to the prevailing “medical model” in which problems and disorders were considered akin to medical diseases and ailments (Rubinstein and Coelho, 1970; Zucker and Gomberg, 1986). Despite the widespread acknowledgment of the importance of biological, psychological, and social factors in contributing to a comprehensive understanding of human life and its problems, there is an increasing awareness that the biopsychosocial model has significant limitations (Pilgrim, 2002; Epstein and Borrell-Carrio, 2005; McLaren, 2007). To some extent the limitations have led to questions regarding whether an integrated bio-psycho-social model has ever eventuated (e.g., Read, 2005).

One of the main problems of the biopsychosocial model is that an actual model that incorporates biological, psychological, and social elements is not currently available. McLaren (1998) conducted an extensive review of Engel’s work regarding the biopsychosocial model and concluded that Engel himself never formulated such a model. Engel strongly advocated for a biopsychosocial model and seemed to have an astute appreciation of what the advantages of such a model would be, however, his work fell short of providing the model he was proposing. The mechanism of negative feedback allows for such a model to be developed. In this paper we will describe Perceptual Control Theory (PCT) which is based on negative feedback and may well be the first biopsychosocial model. Initial work on PCT began in the 1950s and 1960s (e.g., Powers et al., 1960) with the seminal work on this theory first published in 1973 (Powers, 1973) and a second edition published in 2005 (Powers, 2005).

In some ways, it is interesting to consider how the distinction between the biological, the psychological, and the social ever arose. Sir William Osler (1849–1919), who has been described as the father of modern medicine, is reported to have said “It is much more important to know what sort of a patient has a disease than what sort of a disease a patient has”. We are inherently biological entities. A thought is just as much a biological process as is digestion. At the same time, we are also inextricably social. Crucially, our biology would not endure if at least some social interactions (e.g., other people’s supplies of food, shelter, medicine, protection) were not successfully negotiated. We are also undeniably psychological. Our ability to think, reflect, and imagine may be responsible for some of the greatest triumphs of humanity; as well as some of its most devastating tragedies. These separate elements that have been delineated are all in existence, all the time, influencing and affecting each other. It may even be impossible to simply consider individuals as individuals without knowing that each individual has biological, psychological, and social elements that will be important to their successful and ongoing functioning.

Research described by Henrich et al. (2010) powerfully highlights the interconnectedness of the biological, the psychological, and the social. In this research, the IQs of 7-year-old twins from high and low socioeconomic status (SES) backgrounds were assessed. “For high-SES children, where environmental variability is negligible, genetic differences account for 70–80% of the variation with shared environment contributing less than 10%. For low-SES children, where there is far more variability in environmental contribution to intelligence, genetic differences account for 0–10% of the variance, with shared environment contributing about 60%” (Henrich et al., 2010, p. 77).

Regardless of how the separation in the life sciences occurred, fragmentation is very much the status quo. Although some integration between the biological, psychological, and social domains occurs from time to time, it does not appear to be part of a comprehensive and coordinated movement (Pilgrim et al., 2008). An editorial in *The Lancet* suggested that a revolution in psychiatry might be close at hand. This conclusion was based on studies reporting the results of genome-wide analyses indicating that variations in calcium channel activity genes were important in various disorders and could lead to new molecular targets for psychotropic drug development (The Lancet, 2013). Priebe et al. (2013), however, remind us that “neurobiological phenomena are ultimately meaningless unless they are linked to the real lives of people in their social reality” (p. 320).

Brain activity, isolated from the context in which the activity occurs, cannot be understood accurately (Barrett, 2011). Likewise, Cacioppo et al. (2000) assert that “The abyss between biological and social levels of organization is a human construction, however, one that must be bridged to achieve a complete understanding of human behavior” (p. 830). It is our thesis that negative feedback provides the necessary bridge.

In some ways, it might appear that the activity of neurotransmitters crossing a synapse does not have much in common with the activity of colleagues in a work place who are struggling to cope with problems of bullying and intimidation. Our suggestion, however, is that *negative feedback* is a mechanism that is fundamental to the biological, the psychological, and the social domains of human living. Negative feedback may be the glue that does for the biopsychosocial model what all the King’s horses and all the King’s men couldn’t do for Humpty Dumpty—put it together again. Yet, to do so, a precise and accurate definition of negative feedback needs to be used. We will show that although the term is already widely used in the biological and social sciences, the definitions of negative feedback are currently contradictory, vague, or obscured by additional elements within existing models.

It is also important at the outset to be clear about what it is that we are *not* claiming. While we do propose that negative feedback is fundamental to life, we are not suggesting that negative feedback is the *only* process important to living things. The action potential of a neuron, for example, could be understood to propagate down an axon according to *positive*, rather than negative, feedback. Also, *feedforward* has been used as an explanatory mechanism in some areas of functioning. Where other processes such as positive feedback and feedforward are implicated, however, our contention is that these processes are embodied within an overall system of negative feedback. The action potential, for example, occurs within a neuron that is part of a negative feedback system. A system that relied only on positive feedback or feedforward would not function effectively for very long in the unpredictably changing environments that organisms on Earth inhabit. To reiterate, we are not claiming that negative feedback is the only mechanism to be understood but we are proposing that negative feedback is primary and other processes and mechanisms, to be understood accurately, need to be considered within the framework of negative feedback systems.

In this paper we begin by describing negative feedback and its mechanistic nature. Rather than the conceptual or statistical mechanisms that are more common in the biological, psychological, and social sciences, negative feedback is a mechanism in the *functional* sense of the term. After explaining negative feedback and how it works, its importance in the biological, the psychological, and the social spheres will be explained. The embodied nature of this model will also be highlighted. The paper will conclude by suggesting future progress that a field unified and integrated by negative feedback might be able to realise.

## The negative feedback mechanism: what it is and how it works

The concept of negative feedback is not new. Ktesibios, for example, lived in the first half of the third century BC in Alexandria and invented a water clock, which was based on negative feedback principles (Mayr, 1970). By using a float to operate a valve, the water level was regulated by letting more water into the tank when the water level dropped. Ktesibios’s water clock is regarded as the first negative feedback device. Once a more formal understanding of negative feedback was achieved by expressing the important relationships in mathematical terms, its use became more widespread. In particular, the field of control engineering owes its developments to the formalization of negative feedback (Kline, 1993). Today many people rely on negative feedback to keep their home or office at a constant temperature or to keep their car at a constant speed on the motorway.

### What it is

Currently, “negative feedback” is perhaps most commonly associated with criticism about some aspect of a person’s conduct or performance. This is a very different meaning that is not related to negative feedback in the functional sense we describe here. Yet, one hurdle to utilizing negative feedback effectively in research is that the often-published research uses this alternative meaning (e.g., Aguinis et al., 2012).

To understand the functional nature of negative feedback both the words “feedback” and “negative” are important. “Feedback” indicates that output from a system is *fed back* to affect the input of that system. This feeding back process occurs continuously. “Negative” indicates that the effect of feeding the output back to the input is to *negate* or reduce any discrepancy that exists. For example, in a cruise control system in a car, the constant cruising speed is achieved because the speed at which the car is traveling is constantly monitored and any discrepancy between the monitored speed and the set speed (e.g., 55 mph) is reduced.

In a crowded restaurant at lunchtime, a person will speak at a particular volume so that they can be heard by the other people sitting at the table. If there is a sudden hush in the restaurant, the person’s voice will now be too loud and they will quickly reduce the volume of their voice so that they do not seem to be shouting. This reduction happens because the voice they hear (voice output feeding back to auditory input) is now different from the voice they want to hear; so they decrease the volume of their voice to reduce the discrepancy with their desired volume (negative feedback).

To enhance clarity of explanation, it is often useful to illustrate negative feedback with examples from artificial systems such as the cruise control mechanism in cars. There are at least two crucial differences, however, between the negative feedback cruise control system and the negative feedback system that varies voice volume at lunch. The first difference is that for artificial systems the standard is set externally. A cruise control system doesn’t independently decide to move the car along the freeway at 55 mph. For organic systems, however, standards are internally derived. Even if other people at lunch surreptitiously indicate to the speaker that a lowering of voice volume would be appropriate, the volume will only be lowered once the internal standard for volume level has reduced. The second important difference is that a cruise control system controls only one variable—the sensed speed of the car. A speaker at lunch, however, controls many different variables simultaneously. Conversationalists at lunchtime cafes may lower their voices so as not to disturb others but they must also ensure they hold the attention of those to whom they are talking. While they are talking they also need to somehow ensure that they order, eat, and make it back to the office without being late. The usefulness of the simplicity of the cruise control system in explaining concepts in a straightforward manner, therefore, belies the complexity of negative feedback processes in the daily functioning of entities that live.

### How it works

An important but subtle point about negative feedback is demonstrated by the cruise control system. Note that the actual speed that the car travels is *not* determined solely by the output from the engine—that is, how fast the wheels turn. The momentary speed of the car, at every point in time, is determined *jointly* by the output from the engine *and* current environmental factors that also affect the movement of the car such as the gradient and surface of the road or the wind. Similarly, voice volume at lunch is determined jointly by output from the speaker and the surrounding noises of the café. Negative feedback, therefore, describes an ongoing, dynamical interplay between the individual and the individual’s environment. Perhaps it can also be appreciated that a system that monitored only its output and specified how much output to produce, would not be able to create constant effects. If the cruise control system specified a particular *output* that the car should produce then it would travel at different speeds depending on whether the car was going up or down a hill or if there was a strong tailwind or headwind. Also, a person who was able to maintain a constant voice volume throughout lunch would be speaking too quietly on some occasions and too loudly at other times.

The constant effects that a cruise control and a speaker can produce occur because negative feedback is a mechanism that produces *constant input* by *variable output* (Marken and Powers, 1989). If a cruising speed of 55 mph is set, then the cruise control system will vary its output to affect how fast the wheels turn so that the speed that is being monitored stays very close to the set speed of 55 mph. So, output is generated by the discrepancy between the speed that is set and the speed that is monitored. If a standard of “being heard by those at my table” is set, then the speaker’s voice volume will vary so that lunchtime companions will continue to hear what is said (at least from the speaker’s perspective). Importantly, it is the voice, as it is heard by the speaker, that is controlled. A person with a hearing impairment, for example, may be less sensitive to fluctuations in ambient noise levels.

It almost seems as though magic must be involved for a negative feedback system to be able to produce the right amount of output at each moment depending on the particular environmental circumstances that are occurring in order to create a constant result. How could a negative feedback system possibly predict all the different events that could occur and calculate the appropriate amount of output each time so that the state that is being maintained (e.g., 55 mph, voice being heard) is not disturbed?

Actually, it is mathematics rather than magic that provides an understanding of how this occurs and no prediction is necessary. Furthermore, the calculations are surprisingly simple. One equation (*e = r − p*) describes the inside of the system and another equation (*cv = o + d*) describes the outside of the system. This notation depicts the variables that have been described to this point. The set point or desired state of affairs is the reference (*r*); the momentary, continuously monitored value is the perception (*p*); the difference between *r* and *p* is the error (*e*). The error is converted to the output of the system (*o*) by a straightforward amplification constant; *o* combines with environmental disturbances (*d*) to produce the current state of the variable that is being controlled—or the controlled variable (*cv*). To complete the circuit, the *cv* becomes the perception (*p*) by being combined with a slowing constant. By adjusting the values of the amplification and slowing constants the system can be stabilized, oscillations prevented, and a desired state (as specified by *r*) maintained. Thus, the negative feedback mechanism enables the system to control variables in accordance with the set points that have been established. As will be demonstrated, this negative feedback control has important implications in each of the biological, psychological, and social domains.

A prominent theory utilizing a specific implementation of negative feedback is PCT (Powers et al., 1960; Powers, 1973, 2005). From the outset, this approach has been biopsychosocial. In PCT Powers (1973) articulates a neural organization of control systems arranged hierarchically and in parallel that describes the same process of negative feedback occurring across all levels of perceptual complexity from the physiological to the psychological and social. Powers (1973) also uses the robust methodology of model testing through simulations to assess the fundamental tenets of the theory (Marken and Mansell, 2013). Through a careful and detailed explanation of the integration of negative feedback across the range of lived experiences, PCT may be the first functional biopsychosocial model.

PCT, as a model of organismic functioning utilizes a broad applicability of the negative feedback principle. PCT takes the negative feedback control system as the basic building block and develops a model of the systems arranged in parallel and hierarchically. It is not a model only of homeostasis but usefully models the phenomenon of control by negative feedback when set points vary and when multiple systems interact in a network of increasingly complex perceptual control. Berntson and Cacioppo (2007) have provided an excellent review of concepts such as homeostasis, homeodynamic regulation, heterostasis, allostasis, and allodynamic regulation. These concepts and others such as rheostasis (Mrosovsky, 1990) are important for understanding the functioning of organisms and are accommodated within the hierarchical architecture specified by PCT. PCT is an elegant, integrated model in which negative feedback control systems at one level maintain control by varying the references (set points) of lower level systems.

Very often, the efficiency of negative feedback systems is underestimated because their dynamic nature is not fully understood. Across the behavioral sciences, negative feedback systems are used in a sequential manner (e.g., Test-Operate-Test-Exit (TOTE); Miller et al., 1960). Powers (1973) explicitly described a role of sequential processing of the “if … then” constructs described in the TOTE system as occurring at the ninth level (program level) of the eleven level PCT perceptual hierarchy. At lower levels, such as the adjustment of movement as one steers a car to its destination, the process is dynamic and not broken down into stages. In a system such as a TOTE system, however, it seems important to provide additional functions such as “feedforward planning” to make up for the perceived inefficiencies of negative feedback (e.g., Wolpert and Kawato, 1998). Yet, it is our view that this perspective has incorrectly diminished a fundamental property of negative feedback to provide an integration of the biopsychosocial approach. Also, through appreciating the dynamic context in which negative feedback operates *simultaneously* at different levels of the nervous system in its interaction with body and surrounding environment, its embodied nature becomes clear. We will explain these synchronous processes in more detail in subsequent sections.

## Negative feedback at work in the biopsychosocial domain

The hallmark of negative feedback is the maintenance or stabilization of a particular state despite environmental influences that would otherwise alter this state (Powers, 1979). Identifying negative feedback, therefore, entails delineating stabilised states. Negative feedback is revealed by the predictability of these *states*, not the predictability of the actions that achieve them. From this perspective, different actions can achieve the same state. This is because the discrepancy between the actual state and the desired state is of central importance; not just the nature of the desired state itself.

In fact, the importance of the discrepancy between reference states and perceived states may be a critical contribution provided by negative feedback. Goal-directed action, for example, is widely recognized in the literature; however, it has been difficult at times to explain what seems to be the *lack* of a straightforward relationship between goals and actions. Negative feedback clarifies this apparent anomaly by explaining that it is the *discrepancy* between the goal and the currently perceived state that directs actions, not the goal itself. Focusing on the discrepancy between references and perceived states enables observably different actions to be understood in terms of the effects or outcomes they have for the individual.

The psychological process of controlling perception is carried out through a specified organization of neural connections within a physical system (the body and environment). When more than one individual acts within an environment, the social implications are realized, such as the capacity for conflict when reference values differ (e.g., arguments, wars) and collective control when they overlap (the shared values of an organization) (McClelland, 2004).

The psychological model of negative feedback used in PCT is true to the origins of the term in early engineering as we described above. Yet, it is distinct from the ways that other theorists have attempted to integrate negative feedback into psychology. These distinctions appear to have led to a lack of realization of the enormous scientific advances that could be gained from a full understanding of negative feedback in living organisms, to the degree that their models remain strongly influenced by linear cause-and-effect rather than the models of circular causality formed within PCT (Powers, 1989, 1992; Cziko, 2000).

Indeed the influential cyberneticist Ashby (1948) recorded in his journals: “The concept of “negative feedback” is just too simple to be worth anything” (p. 2512) and “The concept of negative feedback is most unsuitable as a fundamental concept” (p. 2524). One of the key “strategic errors” was to place the reference value on the outside, as would be the case for a machine, like a thermostat, that can be set by a human observer. Yet, although mechanical models can be used to explain negative feedback within PCT, all PCT models of living systems ensure that reference values for perception are internally prescribed by the individual.

There also appeared to be a general persistence in cybernetics with the notion that behavior is controlled. Yet, in PCT, it is explicitly perception that is controlled—by behavior, hence the term PCT (Cziko, 2000). Even Powers’ (1973) work on negative feedback has been described in different terms within wide domains of psychology as the “self-regulation of behavior” (e.g., Carver and Scheier, 2001). Yet, this description misrepresents negative feedback in PCT. Powers’ work suggests such a profound difference that people often misinterpret the central tenet (and title of his seminal work) of “behavior: the control of perception” as “behavior: controlled by perception”.

Other explanations have also been put forward for why negative feedback has not reached its full scientific potential, such as the development of digital computers, which made it easier to model linear input-compute-output information processing than the control of continuous variables that occurs within the earlier analog computers of the 1940s and 1950s (Cziko, 2000).

Taking these points together, it appears that although the term “negative feedback” is well used within the biopsychosocial domain, there are strong reasons to suggest that its integrative and applied potentials have been limited precisely because it has typically not been modeled in the way that is required to achieve these insights.

In the following section we will narrow our focus to specific domains of negative feedback, bearing in mind that a description at any level will have implications for the other biopsychosocial domains.

### Biological negative feedback

Negative feedback processes might be most familiar in biological systems. It has been recognised for a long time that the optimum human body temperature is 37.0°C (98.6°F). Actually, a more fine-grained analysis indicates that there is some variability in body temperature depending on the time of day, the place on the body the temperature is measured, and the presence of factors such as infection (Longo et al., 2011). There is also some variability in body temperature between individuals. The variability that has been observed, however, is much less than the variability that would be expected if temperature were only being determined by environmental forces. Indeed, it is largely determined through signals that are internal to the organism (rheostasis; Carpenter, 2004). The neural architecture involved in homeostasis, centered on the hypothalamus, is well documented, and yet outside the work on PCT there is little consideration of whether the remaining systems of the brain implement negative feedback over desired sensory states. When the environment acts upon a rock, the temperature of the rock varies according to changes in the environment. However, when the environment acts upon a person, the person takes action—moves indoors, puts on a coat, opens a window, turns on the air conditioning, perspires—so that their temperature does *not* vary according to the changing temperature of the environment. This is because body temperature is being maintained through negative feedback.

Negative feedback processes have also been identified within the working of neurons. The movement of neurotransmitters in and out of the synapse occurs as part of a negative feedback process (Branco and Staras, 2009). Evidence for this negative feedback activity is obtained when people ingest psychotropic medication that varies neurotransmitter levels. For example, if medication is taken that increases the amount of serotonin that remains in the synapse for a period of time; the body *decreases* the amount of serotonin it releases into the synapse so that the original level is maintained (Wong, 1981). Ironically, it appears to be the case that the reason it can take antidepressants 2–3 weeks before a person notices the medication’s effectiveness is because that is how long it takes for the negative feedback mechanism to become *disabled* (de Montigny et al., 1990). Some medication, therefore, achieves the results it does by thwarting, rather than augmenting, the body’s innate negative feedback machinery (Whitaker, 2010).

### Negative feedback as implemented within neural systems

The use of negative feedback in homeostasis and within the neuron, is relatively well established. However, a third, and arguably more important role of negative feedback, is in the control of sensory input to the brain. As described earlier, this implementation of negative feedback is unique to Powers’ work. Powers (1973) proposed that the core functions required for negative feedback are implemented through known features of neurons. For example, the subtraction function carried out by the comparator within the negative feedback loop is implemented by the convergence of what are considered to be “excitatory” and “inhibitory” connections at the pre-synaptic membrane. An array of other structural arrangements is proposed for other essential functions within a negative feedback system such as addition, multiplication, and integration. Negative feedback necessitates that signaling is bidirectional (both afferent and efferent signals) as there is a simultaneous setting of reference signals through efferent pathways as perceptual signals are fed in through afferent pathways and compared. The functional unit of PCT is therefore an array of neurons that simultaneously pass and transform signals towards and away from the body and environment to keep current perception within desired limits. Often these components of control—neurons, body, social environment—may be widely separated spatially, and yet they form an integrated whole within a dynamic system that maintains desired sensory states.

Within the lower levels of the control hierarchy, Powers (1973) illustrated the neuromuscular pathways of these systems. One example is the tendon reflex loop between higher level reference signals from the central nervous system, spinal motor neurons (acting as a comparator), the effects on muscular contraction, and in turn, the ongoing perception of force carried out by the Golgi tendon receptor and the ongoing perception of position as assessed by annulospiral sense endings. These components act as a complete whole, alongside those elements of the environment that are vital to allow perceived control, such as air for producing speech, and other people who may or may not be perceived to respond as desired by the individual. Thus, the embodied and dynamic nature of this process is clear. This account is necessarily brief, and readers should refer to the original source for this and further pathways, but it should be apparent that the integration of biopsychosocial components through negative feedback is operationally described in examples of this kind.

### Psychological negative feedback

Above, we have proposed that negative feedback at a psychological level is implemented by the neural systems described above. There are many examples of negative feedback within psychology that could be implemented in this way. For example, studies have investigated “set points” and adaptation processes in areas such as happiness and wellbeing. Early research indicated that initial life events such as marriage or divorce had a much smaller effect on levels of wellbeing than might otherwise have been expected. However, later studies have clarified that there is large individual variation in the adaptation that occurs (Lucas et al., 2003). The rate of adaptation also appears to be affected by the positivity or negativity of the event and also whether cognitive wellbeing or affective wellbeing is considered (Luhmann et al., 2012). Negative feedback could provide a framework within which to consider adaptation processes and changes in set point levels.

Mundane concepts such as reinforcement and punishment implicitly invoke the mechanism of negative feedback, although these concepts are rarely discussed in negative feedback terms. A reinforcer, however, essentially has to be something that an organism wants to acquire and a punisher is something an organism wants to avoid. The rats in Skinner’s boxes wanted food pellets under some experimental conditions and wanted to avoid electric shocks under other experimental conditions. In the reinforcement studies, the methodology of Skinner’s work involved “preparing” the rats for the experiments by reducing their weight to approximately 70% of their normal feeding body weight. That meant it was required that the rats be hungry before the reinforcement experiments began (Estes and Skinner, 1941). To put it in negative feedback terms, the rats had an error between how satiated they liked to feel (*r*)** and how satiated they did feel (*p*). In the reinforcement experiments they were given the opportunity to reduce that error (the difference between *r* and *p*) by pressing a bar under various conditions (for example) to receive food pellets and, in other experiments, to avoid shocks (Powers, 1971). The use of reinforcers and punishers will be explored further in the Section Social Negative Feedback.

The application of many clinical psychology treatment programs involves helping people reduce error in their lives. Successful therapy could be defined as a process of enabling people to acquire skills and knowledge so that they can keep important variables in their reference states. Emotions, for example, vary across a range of values and people are often comfortable with certain levels of emotions and uncomfortable with other levels. The field of emotion regulation is based on this approach (e.g., Koole, 2009). Career success, family relationship satisfaction, and involvement in social leisure activities can all be conceptualized as variables. Often psychological distress arises when, for various reasons, people find themselves in a situation where attempting to reduce the error for one important variable actually *increases* the error for another important variable (Powers, 2005). Spending time at work, for example, to achieve a desired level of career success could actually reduce the satisfaction one experiences in family relationships. A range of approaches have proposed that internal conflict underpins psychological distress and that effective psychological treatments either directly or indirectly help people resolve this opposition between important desired states (Emmons and King, 1988; Michalak et al., 2004; Carey, 2008). The concept of conflict from a PCT perspective has strong parallels with the way in which Berntson and Cacioppo (2007) discuss stress disorders arising from allodynamic disturbances with multiple origins in complex interacting systems whereby treatment strategies targeting one dimension may result in “compensatory changes in another dimension that could perpetuate the disturbance” (p. 446).

### Social negative feedback

The use of reinforcers and punishers was mentioned in the Section Psychological Negative Feedback. These techniques have particular relevance in social settings such as schools. Providing the means by which someone can get something they want, or avoid something they do not want, are the hallmarks of reinforcement and punishment. Teachers in schools use stickers and free time, for example, in the hope that students will not have as many stickers or as much free time as they would like, and will follow the teacher’s instructions in order to get more stickers or free time. Similarly, teachers introduce the loss of free time through detentions and suspensions in the hope that students will want to avoid these things and therefore follow the teacher’s instructions.

Understanding negative feedback could improve how systematically and strategically reinforcers and punishers are applied. For example, if a student’s friends are all on detention and this student has a goal of spending time with their friends, the student will experience error between how much time they are spending with their friends and how much time they want to spend with their friends. Students in this situation might use detention as a way of reducing their error. Detention in this instance would seem to not “work” in the sense of producing certain behaviors by being avoided. Seeking to gain a greater knowledge of students’ personal psychological references and the consequent variables they attempt to control in a school environment might help to increase the effectiveness of behavior management programs (Carey, 2012).

An important aspect of clinical psychology treatments is the social or relational component. Negative feedback could enhance our understanding of the therapeutic relationship. Stiles (2009) has described a process of responsive regulation which appears to invoke negative feedback in which therapists vary, for example, the amount of advice they give to clients based on responses from the clients. Thus, therapists have a particular expectation about the way in which they expect to see the clients responding and they vary their behavior to keep the way they are seeing the clients respond matching their expectations. Clients also have expectations about how therapists respond and they vary their behavior to keep the way they are seeing the therapist respond matching their expectations of therapist responding. When considered from this perspective, the therapeutic relationship can be conceptualized as a situation of interacting negative feedback loops (Carey et al., 2012).

The social determinants of health have become increasingly recognised as important in public health interventions since Marmot identified a “social gradient” for risk of heart disease in the Whitehall studies (Marmot, 2006). A fundamental component of the social determinants of health is the phenomenon of control; variously described as the control factor (Tsey, 2008) or “control of destiny” (Syme, 1998). Control is at the heart of negative feedback. In fact, the phenomenon created by negative feedback in which internal states are stabilized against unpredictably varying external conditions is the phenomenon of control (Marken, 1988). The social determinants of health might become even more influential if the negative feedback process inherent in its core constructs was more fully explicated. For example, what is likely to be the critical factor in social conditions affecting health is the extent to which people are able to reduce the gap between their experiences and their expectations in an efficient and ongoing manner. Degrees of freedom in a social rather than a statistical sense are relevant here and refer to the options available to people to keep internal error minimized. Perhaps the degrees of freedom relevant to the mechanism of negative feedback are at the core of the social gradient. As Marmot (2006), p. 565 explains, “What is important is not so much what you have but what you can do with what you have”.

Public health interventions such as behavior change treatments for chronic disease would benefit from greater attention to negative feedback. Although public health programs are delivered broadly, their success hinges on individuals engaging with the material and making the required changes to their behavior. By recognizing the role of behavior in the negative feedback process, public health strategies might become increasingly strategic and systematic by targeting more closely the expectations and internal standards of the individuals who constitute the “public” and providing efficient and effective resources for these individuals to keep their internal errors minimized.

## The importance of negative feedback

Evidence from physical functional models of negative feedback suggests that the negative feedback mechanism and the control it produces are essential to ongoing functioning (Powers, 2005). Tsokolov (2010) points out that an entity that lives will only continue to do so “to the extent that it can compensate for environmental perturbations and reinstate homeostasis” (p. 1033). We have explained that homeostasis is not the only type of regulation that is important to consider when understanding the functioning of organisms. The organization of negative feedback is fundamental to all of biology and should be an essential component of any definition of life (Bourbon, 1995; Tsokolov, 2010). Successful functioning can be understood to arise when barriers to negative feedback are reduced and problems in daily functioning can be considered as disruptions to negative feedback. From a perspective informed by negative feedback it might be possible to understand why treatments are effective when they are and why they fail on the occasions that they do. In the area of cancer prevention and treatment, for example, the success of predicting individual risk based on population level risk factors is moderate at best and knowledge of disease pathways is imperfect (Thrift and Whiteman, 2013). If the findings from statistical studies that made inferences at a population level were combined with model building research that focussed on the performance of negative feedback systems at all levels of embodied functioning for individuals, the accuracy and precision of individual risk prediction might increase exponentially.

At a physiological level it might be possible to begin to develop and administer pharmacological treatments more strategically and systematically so that they enhance rather than disrupt the negative feedback processes that are essential for survival. Consistent with this view, while most pharmacological treatments reduce emotional experiences such as fear, agents that enhance the fear response have been used to augment the beneficial effects of exposure therapy (Hofmann et al., 2011).

At an individual psychological level, knowledge of negative feedback would, once again, allow treatments to be refined and applied more efficiently. Problems of resistance in treatment or lack of insight could be considered anew from a negative feedback perspective. For example, when patients are described as resistant, it would be considered from a patient’s point of view, that the treatment being suggested is disturbing (*d*) one or more reference states (*r*). Time would be well spent, therefore, understanding what these states are and adapting the treatment to suit. Modifications to treatment currently occur in clinical practice, but an understanding of negative feedback would provide a way for treatments to be modified more strategically and efficiently for enhanced effectiveness.

At an interpersonal, social level, negative feedback could inform our understanding of intractable social problems such as conduct disorder and bullying. Social settings such as schools, workplaces, and prisons would benefit from knowledge about negative feedback. The fact that the same reinforcer appears to modify the behavior of some students and not others would be easily and simply explained through negative feedback. Providing computer time as a reinforcer to students, for example, will work for those students who have reference states (*r*) associated with computer usage. For students who prefer other activities, however, computer time will appear to be ineffective as a reinforcer. Similarly, problems such as bullying, truancy, and noncompliance could be understood more clearly by considering internal reference states (*r*), disturbances (*d*), and controlled variables (*cv*). Different students may bully to experience very different perceptual states. Students might want a certain amount of peer approval, or a particular amount of time out of class, or a distinct sense of achievement and satisfaction. Understanding the different states that are being experienced by the same bullying action would allow interventions to be developed that are more meaningful and effective at an individual level.

Negative feedback, therefore, could provide a common language and common concept for researchers and clinicians working on different aspects of the bio-psycho-social individual. From the perspective of negative feedback, clinicians and researchers would understand that the qualifiers bio, psycho, and social simply demarcate different levels of an embodied common process within a complete individual. If the negative feedback mechanism was recognized more explicitly, then common clinical phenomena such as “triggers” could also be understood differently. Rather than triggers being events that “activate” belief systems, which influence attitudes and thoughts and ultimately produce particular behaviors and symptoms of mental health problems; from a negative feedback point of view, “triggers” would be disturbances to perceived states that are being maintained at particular reference levels by negative feedback control systems. Actions occur as a way of restoring these disturbed states to their reference levels; they are not the end result of a linear chain of effects.

The focus for research and practice, therefore, would shift *from* the behaviors and thoughts that individuals produce *to* the perceptual states that are being maintained by these behaviors and thoughts. That is, the perceptual outcomes for the individual of the behavior and thoughts would be emphasized rather than the behaviors and thoughts themselves. By implication, the focus would also shift away from the symptoms of mental health disorders to the distress associated with these symptoms.

Currently, it is difficult to precisely define symptoms of mental health disorders such as delusional beliefs or auditory hallucinations because it is almost always possible to find people in the general population who have the same symptoms but are *not* experiencing mental health problems (Tien, 1991; Peters et al., 2004). It is the distress associated with any particular symptom, however, that is indicative of a mental health disorder not the symptoms themselves (Romme and Escher, 2000). Consequently, research and practice would move from understanding, categorizing, and treating symptoms to understanding and ameliorating psychological distress. The distress would be understood as an inability to reduce error in one or more negative feedback systems with the most common reason for this being the configuration of two negative feedback systems that are in opposition to each other. Reducing error in one system increases the error in the other. Rather than being a deficit model it is actually the reverse in a negative feedback situation. The better the negative feedback systems are, and the more tightly they control, the worse the conflict will be (Powers, 1973; Carey, 2008). Treatments might be able to become more efficient and effective if they are informed by research about negative feedback processes and the ways in which they can be interrupted and also restored to full functioning.

## Integrating negative feedback across the bio, psycho, and social domains: a challenge for future research

While there is good evidence for the existence of negative feedback across the entire spectrum of the lived experience, there is less evidence about how these processes are integrated in a mature, functional system. How does negative feedback at the physiological and biological levels influence negative feedback at the psychological and social levels? A challenge for future research will be to integrate these isolated areas of research to begin to generate accurate descriptions of lives as they are lived (Carey, 2013).

Some of this work has already commenced in terms of understanding the dynamic and constant interaction between the genome and its environment (Zhang and Meaney, 2010). Zhang and Meaney demonstrated that the behavior of mother rats in terms of their licking and grooming of their offspring had dramatic results for the offspring that were evident into adulthood. Mother rats were classified as either high or low in terms of the amount of licking and grooming they did. The offspring of high licking and grooming mothers showed, among other things, “significantly increased hippocampal glucocorticoid receptor expression and enhanced glucocorticoid negative feedback sensitivity” (p. 445). Importantly, when the offspring reached adulthood, they demonstrated “decreased startle responses, increased open-field exploration, and shorter latencies to eat food provided in a novel environment” (p. 446).

Despite these encouraging results, Zhang and Meaney (2010) describe finding ways of conceptually integrating the findings from genetics and psychology as one of our current challenges. Perhaps greater attention to the mechanism of negative feedback and its functioning at different levels of complexity would assist our efforts to achieve meaningful integration. For example, Zhang and Meaney report that cellular signals known as transcription signals regulate the activity of a gene and that environmental signals control the level and activity of the transcription factors. If a gene was understood as a negative feedback control system, however, the environmental signals could be considered as disturbances to controlled quantities of the gene. Changes in the level and activity of the transcription signals might arise through the negative feedback processes of the gene as it reduces the discrepancy introduced by the environmental signals between the set state and the sensed state. Areas such as these could be investigated in future research where geneticists, biologists, and psychologists work shoulder to shoulder to develop an accurate, precise, and integrated account of the process of living.

## Concluding comments

It has been widely accepted for a long time in both research and practice that it is necessary to consider the biological, psychological, and social elements of an individual in order to enhance understanding and treatment. What has been less clear is the way in which these different elements can be integrated into a coherent model that accurately represents a functioning individual. Although an understanding of negative feedback processes has existed for centuries it has only been quite recently that the full extent of the applicability of negative feedback to human living has been appreciated.

The mechanism of negative feedback provides a way of more accurately understanding why effective treatments achieve their effects and also why some treatments fail. By appreciating all human activity as an integral aspect of the process of keeping perceptions of differing complexities at their reference levels, the key elements to target can be better delineated. The focus could shift, for example, from behavioral output to perceptual input. More attention would be devoted to learning about the world from the inside out perspective of the person behaving rather than the external perspective of an observer. The perceptual consequences of behavior would be of greater concern than the behavior itself and distress would receive more attention than the symptoms manifesting the distress.

By understanding the negative feedback mechanism more precisely we may develop a common language and our views about illness, dysfunction, and disorder might change. With a fully integrated biopsychosocial model we may see disciplinary boundaries becoming more permeable, greater advances to our existing knowledge, and far more effective treatments. Through negative feedback we can put the biological, the psychological, and the social pieces of an individual back together again and consider the individual as a complete, fully functioning and integrated individual. Ultimately this may contribute to the maturing of the life sciences with a common physical phenomenon to provide a sense of direction, purpose, and control for the benefit of individuals and societies.

## Conflict of interest statement

The authors declare that the research was conducted in the absence of any commercial or financial relationships that could be construed as a potential conflict of interest.
